# Primary WWOX phosphorylation and JNK activation during etoposide induces cytotoxicity in HEK293 cells

**Published:** 2010

**Authors:** M. Jamshidiha, P. Habibollahi, S.N. Ostad, M.H. Ghahremani

**Affiliations:** 1 Department of Pharmacology-Toxicology, Faculty of Pharmacy; 2 Pharmaceutical Science Research Centre; 3 Department of Molecular Medicine, School of Advanced Medical Technologies, Tehran University of Medical Sciences, Tehran, Iran

**Keywords:** Cell signaling, Cell death, MAPK, Chemotherapy

## Abstract

**Background and the purpose of the study:**

Etoposide is an antineoplastic agent used in multiple cancers. It is known that etoposide induce cell death via interaction with topoisomerase II; however, the etopoisde cellular response is poorly understood. Upon etoposide induced DNA damage, many stress signaling pathways including JNK are activated. In response to DNA damage, it has been shown that WWOX, a recently introduced tumor suppressor, can be activated. In this study the activation of WWOX and JNK and their interaction following etoposide treatment were evaluated.

**Materials and Methods:**

HEK293 cells treated with etoposide were lysed in a time course manner. The whole cell lysates were used to evaluate JNK and WWOX activation pattern using Phospho specific antibodies on western blots. The viability of cells treated with etoposide, JNK specific inhibitor and their combination was examined using MTT assay.

**Results:**

Findings of this study indicate that WWOX and JNK are activated in a simultaneous way in response to DNA damage. Moreover, JNK inhibition enhances etoposide induced cytotoxicity in HEK293.

**Conclusion:**

Taken together, our results indicate that etoposide induces cytotoxicity and WWOX phosphorylation and the cytotoxicty is augmented by blocking JNK pathway.

## INTRODUCTION

Etoposide is an antineoplastic agent with known inhibition of topoisomerase II property which has been demonstrated to have antineoplastic activity in multiple cancers (1) such as acute myeloid leukemia (AML), Hodgkins disease, non hodgkins lymphoma, lung cancer (2), gastric cancer, breast (3) and ovarian cancer (4). Although it is known that etoposide induces cell death via DNA damage due to interaction with topoisomerase II (5), it's cellular response is poorly understood.

Following etoposide induced DNA damage, various cellular pathways including mitogen activated protein kinase (MAPK) are activated (6). The c-jun N-terminal kinase (JNK) is a MAPK which can be activated in response to inflammation, stress, heat shock, UV and growth factors (7, 8). It is shown that JNK has a dual role in cell differentiation and cell death although the exact mechanism is unknown. Three genes encode JNK1, JNK2, JNK3 isoforms with 85% identity among these enzymes. While JNK1 and JNK2 are distributed in most tissues, JNK3 is only present in the CNS (9).

WWOX, an oxidoreductase protein, is a tumor suppressor protein and its defect has been identified in multiple malignancies such as prostate (10), breast (11), lung (12) and gastric cancer (13). It is known that WWOX mediates its effect in response to DNA damage, UV irradiation and staurosporine via increasing p53 stability (7). When WWOX is transiently transfected, 95% of cells died within 3 days. Furthermore, cells transfected with siRNA targeted to WWOX show increase tolerance in response to DNA damage (14) and JNK overexpression inhibits WWOX induced cell death (15). Thus, there is a signaling link between JNK and WWOX with regard to the cell death.

Moreover, the tolerance during cancer therapy results in treatment failure or adverse effects and combination therapy is an effective strategy to avoid drug resistance. Identification of new targets or pathways activated via etoposide gives clues for new combinational therapies. In addition, primary resistance to etoposide in many patients has been reported and understanding the alteration in downstream pathways activated by etoposide will provide new therapeutic approaches. In this study the time course of JNK and WWOX activation in HEK293 cells following etoposide treatment were evaluted. In addition, the viability of the cells treated with etoposide alone or in combination with JNK specific inhibitor was examined.

## MATERIAL AND METHODS

### 

#### Chemicals

(3-[4,5-dimethyl thiazol-2yl]- 2,5 diphenyl tetrazolium bromide (MTT), etoposide and SP600125 were from Sigma (UK); Dithiothreitol (DTT), Western blot detection kit and polyvinylidene fluoride (PVDF) were purchased from Roche applied science (Germany). Phospho-JNK, β-actin antibodies were from Cell Signaling Technology (USA); Phospho-WWOX antibody was from abcam (UK), RPMI-1640, Fetal Bovine Serum (FBS), penicillin-streptomycin, trypsin- EDTA were purchased from Gibco (UK). Biomax film was obtained from Kodak (UK). All other chemicals were from Merck (Germany

#### Cell culture

Human embryonic kidney cells (HEK 293 cells) were obtained from Cell bank of Pasteur Institute of Iran, cultured in RPMI-1640 containing 10% FBS, 1% penicillin-streptomycin and maintained in a humidified atmosphere of 5% CO2 at 37°C. Cells were plated at 106 in 35 mm tissue culture dishes (for protein extraction) or 104 in 96 well plate (for MTT assay) for 24 hrs and treated with 100µM etoposide. Cells were plated triplicates for each treatment group.

#### Western blot

At various time points, cells were lysed for SDS- PAGE and immunoblotting experiments. Prior to lysis, cells were rinsed with ice-cold phosphate buffer saline (PBS), total lysate was prepared using protein lysis buffer (Tris 62.5 mM, pH 6.8, DTT 50 mM, SDS 10%, glycerol 10% and bromophenol blue 0.25%(w/v)) and stored at -80°C. Equal amount of samples were subjected to 10% SDS- PAGE. The gels were then blotted onto PVDF membrane and blocked with 1% casein, 0.05% Tween 20 in TBS at 4°C for 4–6 hrs. The membrane was probed with the 1:1000 dilution of primary antibodies. The membranes then probed with corresponding HRP- conjugated secondary antibody at a 1:10,000 dilution. Signals were visualized using chemiluminescence on Biomax film. Densitometry analysis was performed using Scion Image (Ver. 4.0.2; Scion corporation, USA). The signals obtained for each protein were normalized to β-actin and Mean±SE of three independent experiments were plotted (16).

#### Determination of mitochondrial dehyrogenase activity (MTT)

After drug treatments, cells were incubated with MTT (1mg/ml) for 4 hrs at 37°C. Mitochondrial dehyrogenases of viable cells cleave the tetrazolium ring of the yellow MTT to yield purple formazan crystals which are insoluble in aqueous solutions. The crystals were dissolved in 100 µl of DMSO and the absorbance of the resulting purple solution was measured at 570 nm against 690 nm for blank solution. The amount of produced formazan is directly proportional to the number of viable cells.

#### Statistical analysis

The results were analyzed using one way ANOVA followed by Tukey-Kramer post test. The p value less than 0.05 (p<0.05) was considered significant.

## RESULTS

### 

#### Effect of etoposide on HEK293 viability

**Figure 1:**
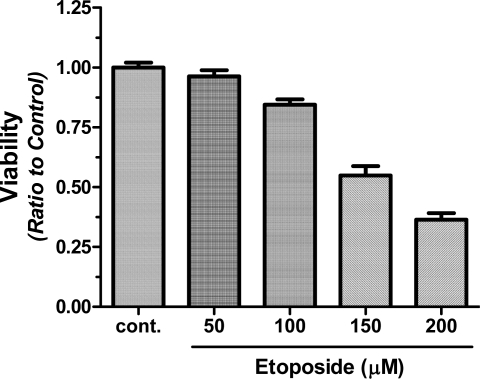
Effect of etoposide on HEK293 cell viability. Cells were seeded at 104 in 96-well plates and treated with etoposide (50,100,150 and 200µM) for 24 hrs and compared to nontreated cells (control). At the end of treatment period, cell viability was measured using MTT assay. OD_570_ were measured and the results were presented as Mean±SD (n=6).

Results of this study indicate that etoposide reduce cell viability at 50 µM by 10%. The cytotoxicity increases to 75% at 200 µM and IC of this effect is about 150 µM (Figure 1). For combination treatments, 100 µM of etoposide was used to observe the synergistic effect better since viability is very low at high doses of etoposide and can mask the synergistic effect.

#### Effect of etoposide on JNK activation

**Figure 2:**
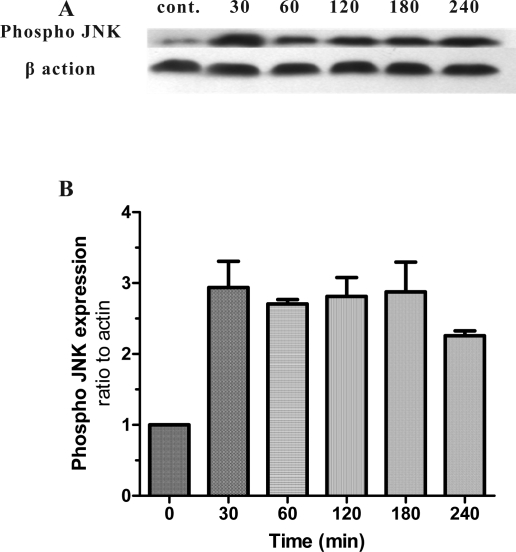
The time course of JNK phosphorylation following exposure to etoposide in HEK 293 cells. Cells were plated 106 in 35 mm dishes and exposed to etoposide 100µM. Total cell lysate was prepared at 0, 30, 60, 120, 180 and 240 min and subjected to SDS-PAGE. **A:** The bands for phospho-JNK and β-Actin were detected by specific antibodies. **B:** The densitometry analysis performed and data presented as Mean±SD of three independent experiments (n=3).

Results also indicate that HEK293 cells have low level of Phospho-JNK in normal conditions. After treatment with etoposide, Phospho JNK level increased to 3 folds of control level (zero time) within 30 min and maintained high during exposure to etoposide (Figure 2). Total JNK expression did not change (data not shown).

#### Effect of etoposide on WWOX activation

**Figure 3:**
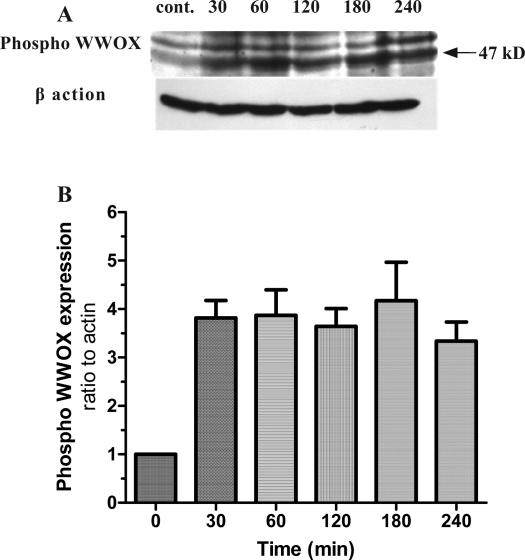
The time course of WWOX phosphorylation following exposure to etoposide in HEK 293 cells. Cells were plated 106 in 35 mm dishes and exposed to etoposide 100µM. Total cell lysate was prepared at 0, 30, 60, 120, 180 and 240 min and subjected to SDS-PAGE. **A:** The bands for phospho-WWOX and β-Actin were detected by specific antibodies. **B:** The densitometry analysis performed and data presented as Mean±SD of three independent experiments (n=3).

Results obtained from Phospho-WWOX indicate that HEK293 cell line has a low level of Phospho- WWOX during normal culture. After treatment with etoposide, Phospho-WWOX increased significantly and reached to 4 fold of zero time and this level was maintained during exposure to etoposide (Figure 3).

#### Effect of etoposide and JNK inhibition on viability of HEK cells

**Figure 4:**
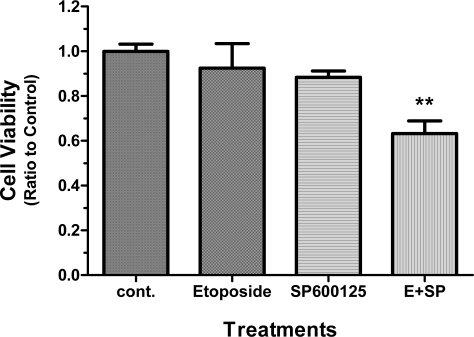
Effect of JNK inhibition on etoposide induced cytotoxicity. Cells were seeded at 104 in 96-well plates and treated with etoposide (100µM) or SP600125 (20 µM) for 24 hrs.The pretreated group (E+SP) received SP600125 for 2 hrs and then treated with etoposide for another 24 hrs. At the end of treatment period, cell viability was measured using MTT assay. OD_570_ were measured and the results were presented as Mean±SD (n=6). The data were analyzed using One way ANOVA followed by tukey post test (** p<0.01 compared to control).

Figure 3 indicates the viability of cells treated with etoposide (100 µM), SP600125 (20 µM, specific JNK inhibitor) and combination of SP600125 and etoposide. As results show, etoposide and SP600125 lowered HEK cell viability after 24 hrs. However, this effect was not statistically significant from untreated cells (control). Interestingly, cells pre- treated with JNK specific inhibitor and exposed to etoposide showed a marked decrease in viability by 50% (Figure 4), suggesting a significant role for JNK function in etoposide induced cell death.

## DISCUSSION

Although DNA damage is the cause of etoposide induced cell death, many pathways are involved in executing cell death. JNK and recently introduced tumor suppressor, WWOX, are proteins that their role to DNA damage has been studied extensively in response (17). Recent studies have shown that cells containing specific mutant forms of WWOX don't respond to DNA damage induced cell death (18). Furthermore, it is known that p53 is activated in response to DNA damage and WWOX siRNA inhibits p53 dependent cell death (14). Thus, cancer cells with defect in their WWOX gene would probably resist to etoposide chemotherapy. Results of this study indicate that etoposide quickly activates JNK and WWOX and the levels of phospho-JNK and phospho-WWOX remain high throughout the exposure. In addition our findings show that the inhibition of JNK synergistically enhances cytotoxicity of etoposide. Therefore, WWOX activation and block of JNK function could have similar effects to exert cell death and could bring up new strategies in specific cancers such as small cell lung cancer. Furthermore, our results indicate that etoposide can increase active form of JNK. Although, it seems that this effect is opposing the cytotoxic outcome of etoposide treatment, it is known that various transcription factors such as c-Jun, ATF-2 and Elk-1 can be activated by JNK (19). Moreover, it has been reported that JNK pathway has a dual apoptotic and anti-apoptotic function (20). Therefore, changes in activation of these transcription factors as well as other signaling cross talks may be responsible for our observations. Furthermore, the synergistic effect of etoposide treatment and JNK inhibition can be mediated via WWOX activation and/or other pathways such as p53. Since DNA damage induced by etoposide utilizes p53 and lack of WWOX protein inhibits p53 dependent cell death, one can propose shared point(s) in JNK and WWOX activation. These shared targets can be related to the cell death pathways involved in these findings. Clearly, further experiments are required to confirm this proposed mechanism. Taken together, results of this study provide evidences which can be utilized to decrease etoposide dose in patients and increase therapy efficiency in resistant cases. It is noteworthy that because of the various effects of JNK in cell physiology, JNK inhibitors can not be a selective therapeutic choice; however, using novel drug delivery systems and/or design of selective inhibitors conjugated to etoposide could be useful in cancer therapy.
